# Use of Right Ventricular Assisted Device for Right Heart Failure in a Patient With Acute Respiratory Distress Syndrome

**DOI:** 10.7759/cureus.17671

**Published:** 2021-09-02

**Authors:** Muhammad U Jahngir, Payam Nabizadeh-Eraghi

**Affiliations:** 1 Internal Medicine, Orange Park Medical Center, Orange Park, USA; 2 Pulmonary and Critical Care Medicine, Orange Park Medical Center, Orange Park, USA

**Keywords:** acute respiratory distress syndrome [ards], right heart failure, impella, mechanical assisted device, pulmonary vascular resistance

## Abstract

Acute respiratory distress syndrome (ARDS) is one of the common etiologies of acute right ventricular dysfunction (RVD) with or without right heart failure (RHF). We present a case of a 40-year-old patient who developed severe ARDS due to massive aspiration of gastric content, secondary to predisposing anatomy of his post-surgical upper gastrointestinal tract. He subsequently developed right ventricular failure. He was treated with a right ventricular mechanical device. Despite all heroic measures, the young patient lost the battle of his life.

## Introduction

The anatomical build of right ventricle (RV) makes it resilient to handle a marked variation in preload (volume-tolerant), at the expense of low functional reserve to acute rise in afterload (pressure-intolerant) [1]. Pulmonary vascular resistance (PVR) is generally one-fourth of systemic vascular resistance (SVR). The sudden increase in PVR can lead to reduced blood flow in the pulmonary vasculature, resulting in increased systemic venous return eventually resulting in right heart failure (RHF). Right ventricular dysfunction (RVD) can be ascertained from echocardiographic evidence of reduced RV systolic function, or unexplained raise of natriuretic peptide in the settings of normal left ventricular function and renal clearance and even RV strain pattern on electrocardiogram (EKG) with or without elevated troponins. RVD is often seen with RHF syndrome [2] and in the settings of cardiopulmonary pathologies (e.g., severe aortic stenosis, acute respiratory distress syndrome (ARDS), acute pulmonary embolism) and has a prognostic value [1,2]. The dilation of right ventricle (either secondary to pressure or volume overload) can press against the inter-ventricular septum, impairing filling of left ventricle (LV) (inter-ventricular-dependence), thus reducing LV stroke volume (SV) and cardiac output (CO) [1]. The low CO and tissue perfusion in the presence of acute RVD is the defining feature of acute right ventricular failure (RVF) [2]. The management of RVF includes optimization of the fluid status, treatment of the underlying cause for raised PVR and titration of inotropic/vasopressor therapy. In refractory cases, more heroic measures, such as interventional or surgical options (i.e., atrial septostomy, RV assist device placement, extracorporeal membrane oxygenation (ECMO), or cardiac transplant) may be employed [3].

## Case presentation

A 40-year-old African American male patient, with a past medical history of chronic achalasia presented with intermittent abdominal pain. He was status-post esophagogastrectomy with colonic interposition and J-tube insertion performed at an outside facility, approximately six months ago. Pain was intermittently present since the time of surgery, moderate intensity, gradually worsening, associated with intractable nausea and vomiting, and located in the left lower quadrant. Patient was well oriented and awake, physical examination was significant for tachycardia and mildly tender left upper quadrant of abdomen. On admission, the computer tomographic (CT) imaging of abdomen/pelvis with contrast showed post-surgical changes and generalized mesenteric edema with no evidence of mesenteric ischemia or internal hernia. Upper gastrointestinal endoscopy was significant for some ulcerative changes at the site of surgical anastomosis, without stenosis and the pathology results were negative for intestinal metaplasia. Through the early course of hospitalization, he was still unable to tolerate diet orally. By hospital day four, he started complaining of blurred right eye vision and horizontal gaze palsy (cranial nerve-VI palsy) was appreciable on physical examination. Computer tomography brain without contrast ruled out intracranial hemorrhage with no evidence of acute pathology. On hospital day six, he was complaining of dizziness that progressively worsened. By the seventh day, his symptoms progressed complicated by confabulation and hallucinations. Cerebrospinal fluid (CSF) examination, as well as magnetic resonance imaging (MRI) of the brain and orbits, were normal.

**Figure 1:**
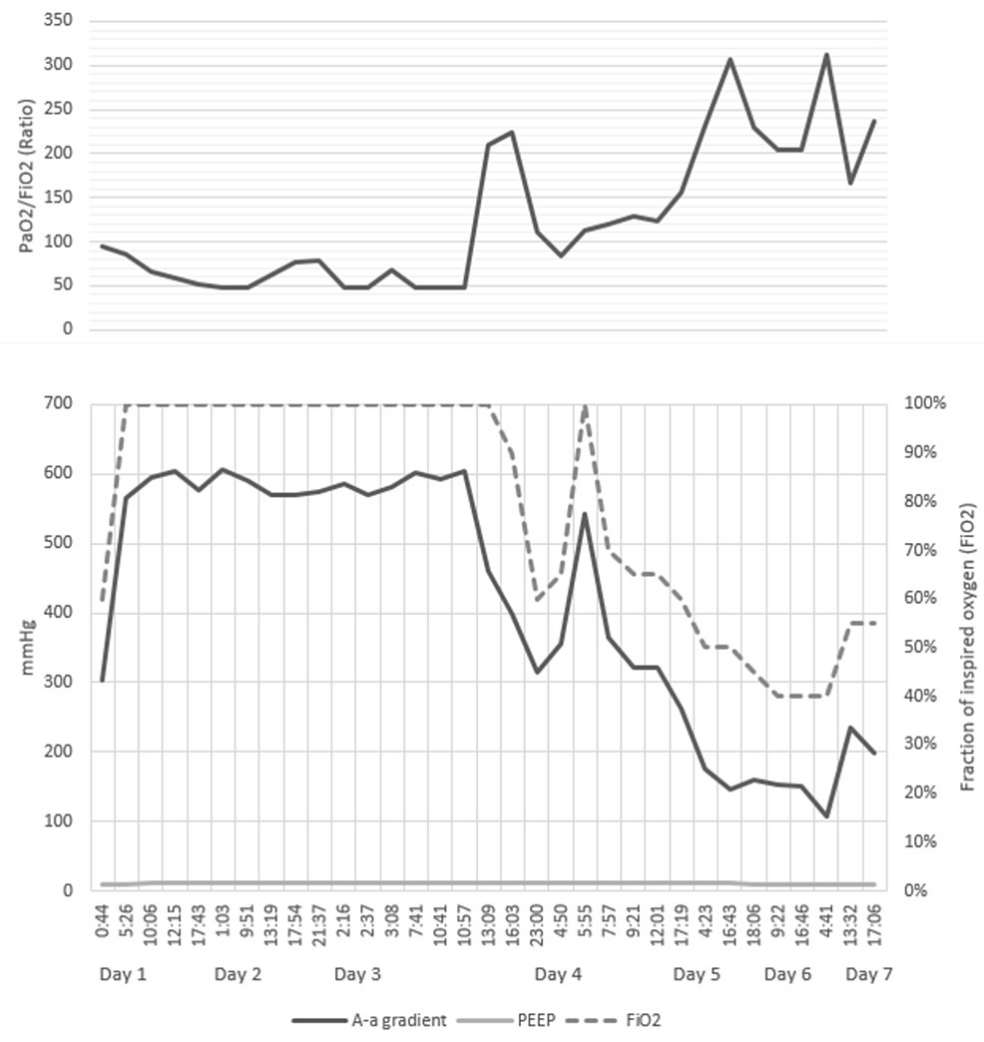
Trend of arterial blood gases (ABGs) and mechanical ventilator settings over the course of ICU admission. PaO2: partial pressure of arterial oxygen; FiO2: fraction of inspired oxygen; A-a gradient: gradient of arterial to alveolar oxygen partial pressure; PEEP: positive end expiratory pressure.

By this time, differential diagnosis included intermittent porphyria and Wernicke's encephalopathy. Urine porphobilinogen was negative; and patient was started on thiamine. On hospital day 9, he was intubated secondary to worsening encephalopathy and inability to maintain his airway. He was then transferred to the intensive care unit (ICU). Of note, the patient had a large volume aspiration event of brownish fluid during the endotracheal intubation. Post intubation ABG was significant for an A-a gradient of 303.8 mmHg and PaO2/fraction of inspired oxygen (FiO2) ratio of 95 while on positive end-expiratory pressure (PEEP) of 8 mmHg. See Figure 1 for more detail of mechanical ventilation from day 1 through day 7 of ICU admission.

Post intubation patient required two vasopressor support (norepinephrine and vasopressin) to keep mean arterial pressure (MAP) as more than 65 mm of Hg. Chest radiograph showed diffuse bilateral patchy opacities consistent with clinical diagnosis of ARDS and computer tomography chest with and without contrast ruled out the pulmonary embolism. Initially, he was treated as distributive shock and covered with broad-spectrum antibiotics. Due to his clinical decompensation and refractory shock, stress dose steroids, intravenous thiamine, and vitamin C were initiated. Repeat echocardiogram in the setting of refractory shock was significant for essentially normal LV function with an ejection fraction of 50-55% but severe RVD with a tricuspid annular plane systolic excursion (TAPSE) of 12 mm. By early next morning, his shock worsened with multiple organ dysfunction syndromes (MODS) requiring four vasopressors (phenylephrine, norepinephrine, epinephrine and vasopressin) to maintain a MAP above 55 mmHg. He was placed on continuous veno-venous hemodialysis (CVVHD) for refractory acidosis in the settings of gradually declining glomerular filtration rate and persistent lactic academia. He was on mechanical ventilation for his severe ARDS (as per ARDSnet protocol) along with pulmonary vasodilatory therapy with inhaled epoprostenol. Due to his severe clinical instability, he was not a candidate for prone positioning or transfer to an extracorporeal membrane oxygenation (ECMO) center.

**Figure 2:**
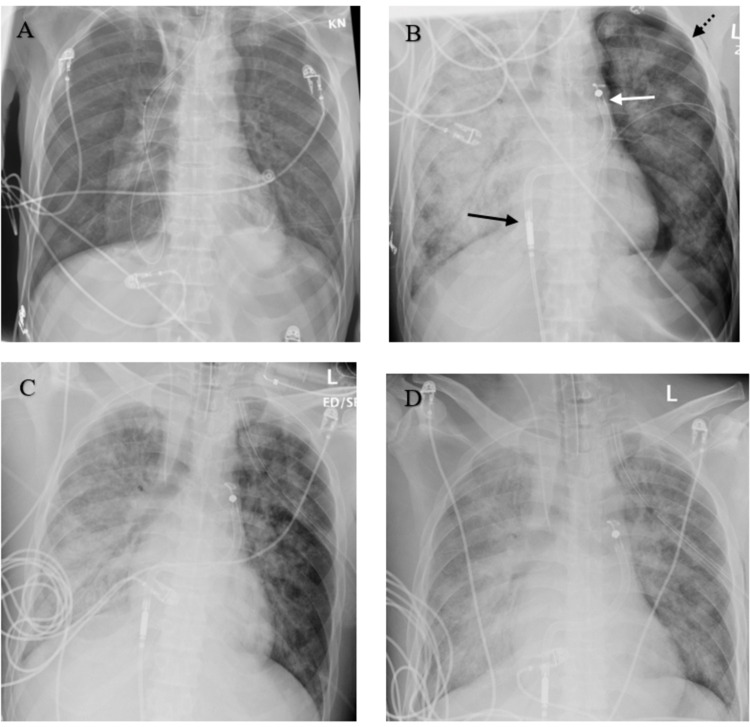
Serial chest X-rays. A. Day 1 of ICU stay ET tube in position. NG tube courses into the right lung. B. Day 3 (am): Right IJ approach central venous catheter with the tip projecting over the SVC. Significant increase in diffuse airspace opacification involving the entire right lung. Impella device visible (black arrow: Inlet area, white arrow: outlet area). Left-sided pneumothorax seen (black arrow with dotted line). C. Day 3 (pm): Interval placement of a left chest tube with the moderate volume left pneumothorax. No pneumothorax appreciated. D. Day 7: No significant interval change with near-complete opacification of the right lung and fluffy opacities throughout the left lung consistent with severe pulmonary edema. Small right pleural effusion.

After multidisciplinary discussion and review of the clinical case, a decision of device placement was made for mechanical support of the right heart via impella. He was transferred to catheterization laboratory and under fluoroscopy, he was found to have a new left-sided pneumothorax (Figure 2B), not evident on last chest imaging (Figure 2A). It was attributed to barotrauma secondary to bag-valve-mask ventilation, required during his transportation to the catheterization laboratory. The event was followed by chest tube placement with complete resolution of pneumothorax. Immediately after the impella placement (Figure 2B), the need for vasopressors was significantly reduced by nearly 50%. The option to transfer him to ECMO center was re-considered, yet he was not deemed a candidate due to his multi-organ dysfunction and number of days on the ventilator.

He continued to have significant improvement in both hemodynamics and multiorgan dysfunction. Vasopressor support, as well as ventilatory support, decreased to a minimum in 48-72 hours post right heart impella placement. Unfortunately, he did not have a significant improvement in his neurological status.

**Figure 3:**
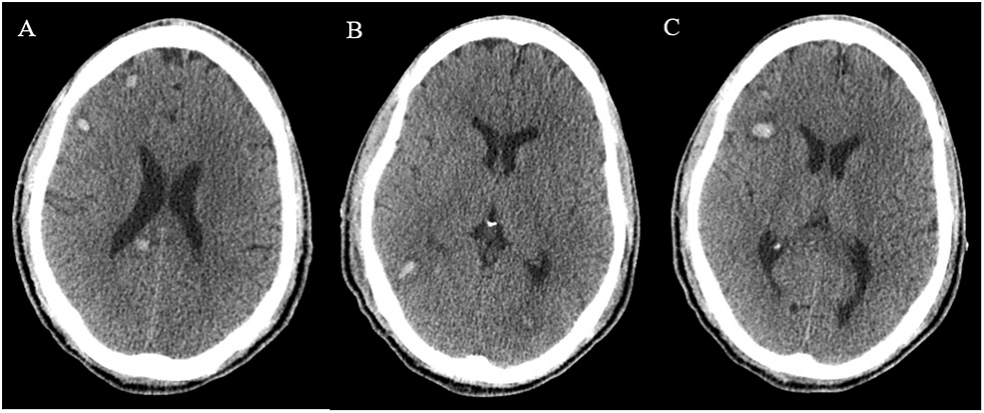
Computer tomography (CT) brain. Multiple foci of acute parenchymal hemorrhage involving both cerebral hemispheres mostly at the gray-white junction, with associated surrounding edema (A, B). The majority of the lesions measure under 1 cm with the dominant lesion in the right frontal lobe (C).

**Figure 4:**
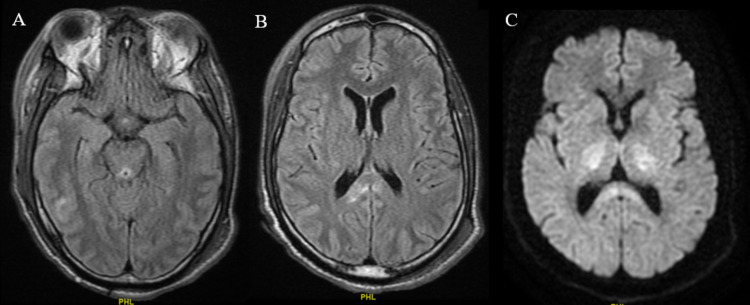
Magnetic resonance imaging (MRI) brain. A. T2-weighted Fluid-Attenuated inversion recovery (T2 FLAIR) image: Increased signal within the periaqueductal gray matter and extending along the dorsal aspect of the brainstem. B. T2 FLAIR image: Hyper-intense signal within the right greater than left splenium of the corpus callosum. C. Diffused-weighted (DWI) image: Subtle restricted diffusion and expansion of the posterior limbs of each internal capsule.

The repeat CT brain (Figure 3) was consistent with cerebral edema and multifocal hemorrhages in bilateral cerebral hemispheres, with the largest lesion measured approximately 1 centimeter. Possible etiologies included systemic anticoagulation while on CVVHD and mechanical cardiac device, coagulopathy in the settings of shock liver, thrombocytopenia secondary to increased consumption, and possibly compounded by probable thrombasthenia as a sign of worsening uremia. On hospital day 15, he was down to just one vasopressor (vasopressin) and impella was removed on the sixth day of its placement. MRI brain without contrast was performed and was suggestive of extensive damage consistent with Wernicke-Korsakoff’s syndrome (Figure 4), with superimposed multifocal hemorrhagic strokes (This was previously deferred while Impella in place).

Given the global extent of the underlying neurologic pathology, the patient’s family decided to transition to comfort measures. The patient died soon after weaning off the mechanical ventilation.

## Discussion

The reported incidence of acute RVD in patients with ARDS is 30%-56%, with increased 28-day mortality [2]. Pneumonia as a primary etiology, PaO2/FiO2 ratio of < 150 mmHg, PaCO2 of ≥ 48 mmHg, and driving pressure (plateau pressure - PEEP) of ≥ 18 mmHg, are four predictors of acute RVD in these patients [4]. Echocardiographic parameters for RVD are given in Table 1.

**Table 1:** Assessment of right ventricular function on echocardiogram [1,2,5]. EDD: end-diastolic diameter; EDA: end-diastolic area; LV: left ventricle; PASP: pulmonary artery systolic pressure; RV: right ventricle; TAPSE: tricuspid annular plane systolic excursion; TTE: transthoracic echocardiogram; TEE: transesophageal echocardiogram; #peak velocity of systolic excursion at the lateral tricuspid annulus; *prognostic value.

Parameters	Interpretations	Modality/view
Signs of RV dilation		
Basal RV diameter	> 42 mm	TTE: apical four-chamber; TEE: mid esophageal four chamber
RV mid diameter	> 33 mm	
RV/LV EDD	> 0.9	
RV/LV EDA	> 0.6	
Septal dyskinesia (D-shaping)		
Systolic & diastolic phase	RV pressure overload	TTE: apical four-chamber or parasternal short-axis view at the end-systole and end-diastole
Isolated diastolic phase	RV volume overload	
RV wall thickness	>5 mm	
Signs of RV systolic dysfunction		
TAPSE	<16 mm	TTE/TEE: (TAPSE) M-mode imaging at the lateral tricuspid valve plane Swan Ganz Catheter (PASP)
TAPSE/PASP*	<0.36 mm/mmHg	
S wave#	< 10 cm/s	TTE: apical four-chamber; TEE: deep transgastric view of RV
RV Ejection fraction	<45%	TTE/TEE: RV focus four-chamber view
RV fractional area change	<35%	
Peak RV free wall 2D strain	>-20%	

Temporary mechanical support of RV can hasten the recovery in some patients [6]. Over the past two decades, a number of mechanical ventricular devices have been developed to support acutely decompensating right heart. The working mechanism of these catheter-based devices, is to connect the inflow tract directly to the outflow tract [7]. Impella works on the same principle and is composed of microaxial pump including a drive motor and an impeller [7] that sucks the blood from inferior vena cava (IVC) and pumps it into the pulmonary artery. The device induces direct augmentation of pulmonary artery flow with simultaneous decrease of the right heart preload. It not only reduces the need for inotrope/vasopressor support by decreasing systemic venous congestion and improving the end-organ perfusion [8] but also decreases the myocardial oxygen consumption by improving subendocardial blood flow and decreased wall stress [9]. 42% to 75% of the patients with acute RVF recover their hemodynamics followed by device implantation [6].

The impella placement is primarily studied in the critically ill patients who had right ventricular failure after cardiothoracic surgery for cardiotomy, secondary to acute myocardial infarction or after the left ventricular assisted device (LVAD) placement [6,8,10]. Food and Drug Administration (FDA) first approved impella for bridge-to-recovery support in the patients with acute RVF on September 20, 2017 [10]. Although, the first-ever right heart impella was implanted back in 2013, through the minimally invasive catheter-based approach in the United States, in a 64 years-old male patient with refractory cardiogenic shock secondary to inferior wall myocardial infarction. The device was explanted after six days with significant clinical and radiological improvement of RV function [11].

Cheung et al. [6] conducted a retrospective cohort study, showed promising results for acute refractory right heart failure by significant improvement in cardiac index immediately after the device implantation for an average duration of seven days. 44% of the patient revealed normal RV function, while 44% of the patient had mild to moderate residual right ventricular dysfunction on one-year follow-up echocardiograms.

The RECOVER RIGHT study, which was a prospective cohort of 30 adult patients, has further reinforced the fact of immediate hemodynamic improvement after the placement of right heart impella, where patients were supported for a mean duration of 3.0 ± 1.5 days. The study included the patients with cardiogenic shock secondary to right ventricular failure (with cardiac index (CI) between 1.3 - 2.2 L/min/m^2^) despite being on inotrope(s)/vasopressors with central venous pressure (CVP) of >15 mmHg, and right ventricular dysfunction evident on echocardiogram (TAPSE ≤ 14, RV diameter of >42 mm at base) or right heart catheterization (CVP/pulmonary capillary wedge pressure (PCWP) ratio of >0.63). This group of patients did not include those with critical organ dysfunction, right heart or pulmonary artery thrombus, severe pulmonary artery hypertension and those who already had right heart mechanical devices or valves in place. The overall six-month survival was 70% [8].

Another single centered retrospective study was published from Denmark, where the impella devices were placed in 109 patients over the course of four years. The primary indication for mechanical device placement in this population was myocardial infarction with refractory cardiogenic shock. The results were convincing, where the benefits of impella device placement in acute settings of heart failure outweigh the complications [9]. Despite triaging the patients as recommended [12], one out of four patients died while on mechanical support, primarily secondary to multi-organ dysfunction. They reported lactic acidosis, to be the strongest predictor of early deaths in these patients [9]. Pappalardo et al. have reported biventricular impella placement (Bi-pella approach) for the first time in a young patient with encouraging results in the acute settings. However, the follow-up results were not given [13].

A recently published Manufacturer and User Facility Device Experience (MAUDE) database give the most comprehensive insight to the usage and adverse effect profile for the placement of right heart impella. The most prevalent reported complication was bleeding (42.9%) with 80% of the patients requiring blood product transfusion, followed by vascular complications (22.8%) with 62.5% requiring surgical repair [10]. Bleeding as one of the complications must be confounded by the fact that patients were already coagulopathic secondary to refractory shock state, congestive hepatopathy [8,10] and being on systemic anticoagulation while device in place [8, 10, 13]. Other complications include; hemolysis [7,8], device malfunction, and in-device thrombosis [10]. After evaluation of the most recent post-approval study (PAS) results, FDA warned health care providers about the increased mortality in patients receiving right ventricular impella system, in the subgroup of PAS patients who were outside the bracket window defined in premarket clinical studies [14].

Lastly, we are hypothesizing the fact that in our patient with failing heart, nutritional deficiency likely played a role. Wernicke Korsakoff's syndrome has been well associated with thiamine deficiency, but the published literature has emphasized the possible contribution of thiamine (vitamin B1) deficiency in heart failure [15-18] and rarely in reversible severe pulmonary hypertension [19]. Thiamine deficiency decreases the production of ATP and increases cellular adenosine, in turn causes decreased systemic vascular resistance and high cardiac output (high output failure), that finally exhausts into myocardial dysfunction and decreased cardiac output [18]. Thiamine is essential for cellular growth and development, as its biologically active form (thiamine pyrophosphate) is a cofactor to many enzymes in cellular metabolism [15]. The half-life of thiamine is 10-20 days and acute vitamin deficiency has been reported in severe catabolic states including sepsis. Testing for serum levels has its limitations, therefore erythrocyte transketolase activity (ETKA), a functional assay, is more precise measurement of thiamine levels [18] but cost, availability and abnormal hemoglobin levels can raise a question.

## Conclusions

Acute RHF is common in the patients admitted to the intensive care unit with ARDS, irrespective of its primary etiology. Acute RHF can be managed with a right ventricular assisted device or ECMO as a bridge therapy until the patient recovers through acute heart failure or receive a cardiopulmonary transplant.
